# De Novo Functional Characterization of AcABI5 Transcription Factor and Its Role in Physiological Responses to Salt Stress in *Alhagi camelorum* Callus

**DOI:** 10.3390/ijms27093812

**Published:** 2026-04-24

**Authors:** Zhengtao Yan, Ya Zhan, Xiangyi Li, Bo Zhang, Gangliang Tang

**Affiliations:** 1 State Key Laboratory of Desert and Oasis Ecology, Xinjiang Institute of Ecology and Geography, Chinese Academy of Sciences, Urumqi 830011, China; yanzhengtao23@mails.ucas.ac.cn (Z.Y.); 17337145997@163.com (Y.Z.); lixy@ms.xjb.ac.cn (X.L.); zhangbo@ms.xjb.ac.cn (B.Z.); 2 Xinjiang Key Laboratory of Desert Plant Roots Ecology and Vegetation Restoration, Xinjiang Institute of Ecology and Geography, Chinese Academy of Sciences, Urumqi 830011, China; 3 Cele National Station of Observation and Research for Desert-Grassland Ecosystems, Cele 848300, China; 4 University of Chinese Academy of Sciences, Beijing 100049, China; 5 National Engineering Technology Research Center for Desert-Oasis Ecological Construction, Xinjiang Institute of Ecology and Geography, Chinese Academy of Sciences, Urumqi 830011, China

**Keywords:** *Alhagi camelorum*, salt stress, callus induction, *AcABI5*, bZIP transcription factor, antioxidant enzymes

## Abstract

*Alhagi camelorum* is a dominant leguminous shrub distributed in the Taklamakan Desert, an area characterized by extreme drought and high soil salinization, which can complete its life cycle normally in salt-affected soils. However, the underlying molecular regulatory mechanism of its salt tolerance remains largely unclear. The *AcABI5* gene was successfully cloned and characterized, and it encodes a typical nuclear-localized bZIP transcription factor. Functional characterization demonstrated that overexpression of *AcABI5* markedly improved the salt stress tolerance of *A. camelorum* calli, whereas silencing of *AcABI5* via virus-induced gene silencing (VIGS) rendered the plant more sensitive to salt stress. Further mechanistic investigations revealed that *AcABI5* enhanced salt tolerance by regulating the expression of superoxide dismutase (SOD)- and peroxidase (POD)-related antioxidant genes. Compared with the wild type, *AcABI5*-overexpressing calli exhibited significantly increased SOD and POD activities and remarkably reduced malondialdehyde (MDA) content under salt treatment, whereas *AcABI5*-silenced lines exhibited the opposite physiological phenotypes. Furthermore, heterologous silencing of *AcABI5* in *Nicotiana benthamiana* via virus-induced gene silencing (VIGS) produced comparable salt-sensitive phenotypes, similar to those observed in *A. camelorum AcABI5*-silenced lines. Collectively, these results provide insights into the molecular mechanism by which *AcABI5* enhances salt tolerance in *A. camelorum*, and lay a solid theoretical foundation for the optimization of the *A. camelorum* genetic transformation system and the expansion of related salt-tolerant crop research.

## 1. Introduction

**Figure 1:**
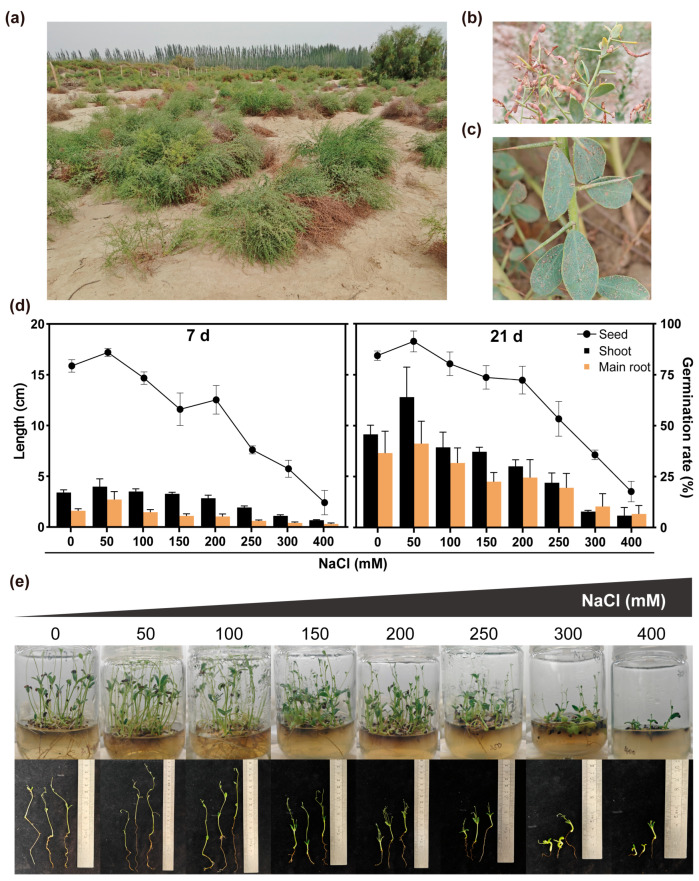
Effects of NaCl on the growth and morphology of *A. camelorum*: (**a**) Representative photograph of *A. camelorum* growing vigorously in desert habitats, demonstrating its adaptation to arid and saline–alkali environments. (**b**) The fruit of *A. camelorum*. (**c**) Leaves and thorns of *A. camelorum*. The images were taken in the southern margin of the Taklamakan Desert. (**d**) The data of the plant height, root length, and germination rate of *A. camelorum* seeds at 7 and 21 days of germination under 0–400 mM NaCl stress are presented (*n* = 3, means  ±  SD). (**e**) The figure shows *A. camelorum* seedlings grown on MS media with different salt concentrations for 21 days.

Soil salinization, exacerbated by climate extremes and land misuse, imposes ionic toxicity and osmotic stress that globally curtail root elongation, biomass allocation, and crop yield potential [1]. Shrinking freshwater reserves now make the reclamation of saline–alkali land a critical imperative for global food security. *Alhagi camelorum* Fisch., a deep-rooted perennial legume of Central Asian deserts, forms 40–130 cm thickets of thorny, gray-green shoots that carry obovate leaves and short axillary racemes whose red-purple petals give way to spiraled loment pods [2,3]. *A. camelorum* spans the arid corridor from the Taklimakan to the Iranian Plateau, occurring in northwestern China, Kazakhstan, Afghanistan, Iran, Pakistan, Iraq, Mongolia and India; across these regions, it routinely colonizes saline soils with >200 mM NaCl, making it one of the most salt-tolerant wild legumes (Figure 1a–c) [4,5,6].

On the southern rim of the Taklamakan, *A. camelorum* carpets the shifting ecotone between oasis cropland and drifting dunes, forming the keystone thicket that stabilizes soils and defines the desert margin [6]. A single stand delivers a triple dividend: its thorny lattice brakes the wind and anchors shifting sand [7], its shoots furnish protein-rich fodder equal to alfalfa [8], and its resin-rich exudates remain a staple of traditional pharmacopeias [3,9,10], and is also an excellent candidate plant for saline–alkali land remediation [11]. Nevertheless, the molecular determinants enabling *A. camelorum* to maintain growth under saline conditions remain largely uncharacterized. Mining and validating potential salt-tolerant genes from *A. camelorum* contributes to improving the salt tolerance of crops and fostering new varieties via molecular breeding techniques, which holds great scientific and practical significance.

In response to complex environments and various stresses, plants have evolved an intricate and extensive molecular regulatory network [12]. Transcription factors play a pivotal role in plant responses to abiotic stresses such as salt and drought stress, as they can regulate the expression of a series of genes associated with these stress responses. Some transcription factors also act in conjunction with other transcription factors to combat abiotic stresses; for example, the *Arabidopsis* NAC transcription factor ANAC096 interacts with bZIP-type transcription factors to jointly participate in drought and osmotic stress responses [13]. *TaABI5* and *TaICE1* cooperatively regulate cold tolerance in wheat under low-temperature stress [14]. MdMYB63 interacts with MdERF106, and together they regulate anthocyanin biosynthesis, mitigating damage caused by salt stress [15]. The *Arabidopsis* response regulator ARR18 negatively interferes with the transcriptional activity of bZIP63 on the *PDH1* promoter, thereby mediating the regulation of proline dehydrogenase expression. Transcription factors regulate the expression of downstream genes to synthesize or degrade specific proteins, enzymes and metabolites, which constitute the defense mechanism of plants against both biotic and abiotic stresses [12].

The basic leucine zipper (bZIP) transcription factor family is the largest, highly conserved and functionally diverse group of transcriptional regulators in plants. They are characterized by a conserved bZIP core domain that encompasses a DNA-binding region and a leucine zipper dimerization domain. Extensive research has confirmed that bZIP transcription factors play a crucial role in plant responses to abiotic stress and in growth and development [16,17]. Following post-translational modifications, these transcription factors participate in protein assembly, regulate downstream gene networks, or interact with other transcription factors. The bZIP family plays a central role in the growth, development, and stress adaptation of leguminous plants [18,19,20]. For example, *GmTRAB1* significantly enhances soybean (*Glycine max*) drought tolerance. Upon silencing *GmTRAB1* in soybean hairy roots, soybeans become more sensitive to drought [20]. *GmbZIP60* in soybean can be induced by salt and drought stresses; overexpression of *GmbZIP60* enhances soybean tolerance to salt and drought by directly binding to the promoters of stress-responsive genes [21]. Researchers pointed out that the model plant *Arabidopsis* bZIP family consists of 78 members, which are grouped into 13 clusters (A, B, C, D, E, F, G, H, I, J, K, L and S) [22,23,24]. Notably, Group A members have been confirmed to exert pivotal roles in plant responses to diverse environmental stresses. Most of them not only participate in regulating plant tolerance to abiotic stresses, including cold, drought and salt stresses, but also are closely associated with the abscisic acid (ABA) signal transduction pathway [25]. Recent studies have shown that Group A bZIP transcription factors in *Arabidopsis* include ABI5, ABF1, ABF2/AREB1, ABF3, and ABF4, which participate in ABA-regulated and stress-induced gene expression [26,27,28,29,30]. Among these, abscisic acid-insensitive 5 (ABI5) is the best-studied key molecule in the ABA signaling pathway [31,32,33]. For example, QKY interacts with ABI5 and promotes its cytoplasmic degradation via the E3 ubiquitin ligase KEEP ON GOING (KEG), thereby facilitating the recruitment of ABI5 to phase-separated condensates. When the QKY function is lost, seeds become more sensitive to ABA, and germination rates consequently decrease [34]. PavABI5 directly binds to the abscisic acid-responsive element (ABRE) within the cherry cold-inducible gene (PavCIG1/2) promoter, activating its expression, and their gene expression levels are positively correlated [35].

As a dominant sand-fixing plant in arid and semi-arid saline–alkali regions, *A. camelorum* plays a central role in improving regional soil structure and maintaining ecosystem stability by virtue of its excellent salt and drought tolerance. Due to its unique ecological value and strong salt tolerance, existing studies have revealed the salt-tolerant mechanisms of *A. camelorum* at physiological, biochemical and molecular levels. Physiologically, it adapts to salt stress through specialized ion compartmentation and selective transport, enhanced antioxidant enzyme activities, accumulation of osmotic regulators, and tolerant and adjustable biological nitrogen fixation [2,36]. At the molecular level, salt stress induces upregulated expression of key genes involved in proline synthesis and flavonoid biosynthesis, accompanied by significant accumulation of corresponding metabolites [37]. As a member of the bZIP transcription factor family, ABI5 plays a critical role in multiple physiological processes during plant growth and development. To date, the biological functions of ABI5 family members in *A. camelorum* have not been systematically investigated. Accordingly, this study was conducted to elucidate the mechanism by which ABI5 regulates salt tolerance-related genes in *A. camelorum*, thereby filling the current research gap in this field.

Calli grow rapidly and are easy to manipulate, making them widely employed in scientific research, especially for investigating the mechanisms underlying plant responses to abiotic and biotic stresses. Here, we developed an in vitro callus system to dissect this trait without soil heterogeneity. In this work, aseptic seedlings of *A. camelorum* served as the experimental materials, and young stem explants were subjected to a hormone matrix screening that yielded a single optimized medium supporting rapid, highly synchronous callus proliferation. Previously, Tang et al. [38] conducted transcriptomic analysis and demonstrated that a series of differentially expressed genes (DEGs) in *A. camelorum* were significantly induced and upregulated under osmotic stress. Through quantitative real-time PCR (qRT-PCR) validation and screening, we cloned the *AcABI5* gene with a 1080 bp CDS from these candidate genes. Based on the above background and preliminary results, we hypothesize that *AcABI5* enhances salt stress tolerance in *A. camelorum* by regulating the expression of antioxidant genes to alleviate oxidative damage under salt conditions. Our study revealed that AcABI5 exerts a positive regulatory role in modulating the salt stress tolerance of *A. camelorum*. Combined with the results of yeast one-hybrid (Y1H) assays on the cis-acting regulatory elements, we initially elucidated the AcABI5-mediated regulatory mechanism underlying the salt stress response in *A. camelorum*. These findings provide new insights into investigating the abiotic stress response mechanisms of desert plants, offering potential for the rational development and application of salt-tolerant varieties.

## 2. Results

### 2.1. Screening of Salt Stress Concentrations and Optimization of Medium Formulation for Callus Proliferation

The *A. camelorum* plants exhibited distinct phenotypic alterations in response to NaCl-induced salt stress. The growth of *A. camelorum* seeds on MS medium with 0–400 mM NaCl for 21 days is shown in Figure 1d,e. Seeds germinated even at 400 mM NaCl, but salt concentrations markedly affected seedling growth: the plant height, root length and germination rate all increased first and then decreased with rising NaCl concentration, with the 50 mM group showing the highest values on days 7 and 21, indicating that low NaCl concentrations promoted seedling growth. At 21 days, these indices declined gradually in 50–200 mM NaCl with a slowing decrease rate. Tillering appeared at ≥150 mM NaCl (obvious stress), germination rate dropped significantly at 250 mM NaCl (seedlings remained bright green with a few showing basal swelling), and severe maldevelopment (abnormally swollen cotyledons, inhibited root elongation) occurred at 300 and 400 mM NaCl, with germination rate falling to 17.67% (severe stress). Based on previous studies [2] and combined with the above results, 200 mM NaCl induced a significant salt stress response in *A. camelorum* without severe growth inhibition; thus, the concentration was selected for the present study.

A broad spectrum of auxin–cytokinin regimes (Table S1) induced callus formation from *A. camelorum* stem explants, with marked variations in macroscopic texture, pigmentation and growth kinetics (Figure S1). Auxin-to-cytokinin ratios ranging from 3:1 to 2:1 consistently produced compact, fast-proliferating callus in *A. camelorum*. Accordingly, 1.5 mg/L 2,4-dichlorophenoxyacetic acid (2,4-D) combined with 0.5 mg/L 6-benzylaminopurine (6-BA) was set as the benchmark for all subsequent experiments (Group 27).

### 2.2. Identification and Bioinformatics Analysis of the bZIP Transcription Factor AcABI5

**Figure 2:**
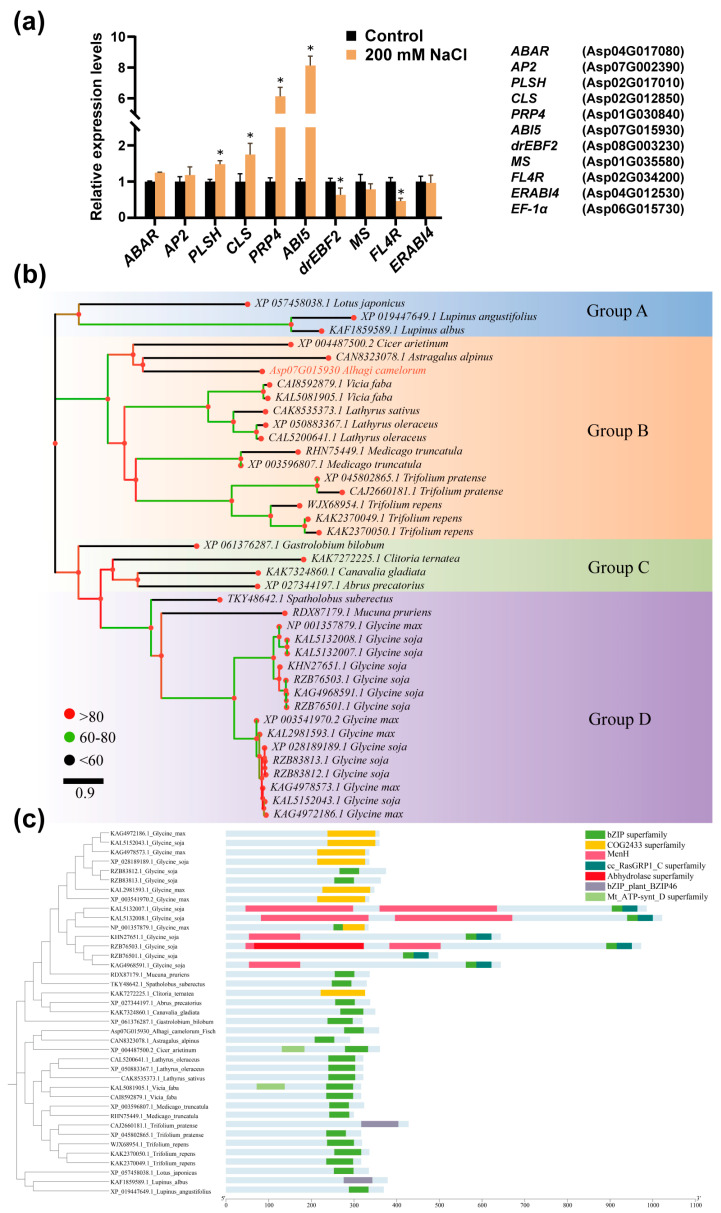
Identification and bioinformatics analysis of AcABI5: (**a**) The relative expression levels of different genes in response to salt stress in *A. camelorum* calli were determined at 6 h after salt stress treatment using *EF-1α* as the reference gene (means  ±  SD, *n* = 3; *, *p* < 0.05; Student’s *t*-test). (**b**) Phylogenetic tree constructed based on ABI5 homologous proteins identified from *A. camelorum*, *M. truncatula*, *C. arietinum* and other plants. (**c**) Conserved domain and motif analysis was performed on ABI5 homologous proteins identified from *A. camelorum*, *M. truncatula*, *C. arietinum* and other plants. The light blue background bars represent the full-length amino acid sequences of the proteins. The colored blocks indicate conserved domain superfamilies at their corresponding positions, as defined in the legend.

We performed qRT-PCR analysis on ten candidate genes [38], and four were significantly upregulated in *A. camelorum* calli under salt stress (Figure 2a). Among these, *ABI5* (Asp07G015930) showed the maximum upregulation (8-fold), and thus was selected as the target gene for subsequent studies.

Based on transcriptome data [38], the CDS length of *ABI* (Asp07G015930) is 1080 bp, encoding 359 amino acids. The CD-search analysis using the NCBI-CDD showed that this protein contains a typical bZIP conserved domain with an E-value of 1.54659 × 10^−7^ and a bitscore of 47.69. This domain is highly conserved in ABI5 homologs from various legume plants, including *Medicago truncatula*, *Cicer arietinum*, *Astragalus alpinus* and *G. max*, indicating high homology with known ABI5 proteins (Table S6). Based on its function and sequence characteristics, Asp07G015930 was named *AcABI5*.

Phylogenetic tree reconstruction was performed using 38 homologous sequences from plants such as *M. truncatula* and *C. arietinum* obtained by homologous protein alignment of AcABI5. AcABI5 is localized in the Group B branch of the phylogenetic tree and is closely clustered with homologous sequences from species such as *A. alpinus* and *C. arietinum*, forming a legume-specific subclade with high support (Figure 2b). This branch forms a clear differentiation boundary with multiple *G. max* sequences in Groups C and D, indicating obvious species differentiation of AcABI5 within legumes.

According to the protein domain analysis, the AcABI5 protein possesses a typical bZIP TF domain located in the proximal C-terminal region of the protein, consisting of a basic region with the conserved motif N-X7-R/K and a leucine zipper region with the conserved motif L-X6-L (Figure 2c and Figure S2). This domain serves as the core functional region determining DNA binding and dimerization. A small number of COG2433 and Men1-related motifs are embedded at both ends, which are highly consistent with other legume homologous proteins. The presence of the bZIP domain indicates that this protein may bind to cis-acting elements to regulate the transcription of downstream genes and play a key role in plant stress responses (such as drought and salinity). In addition, the PlantCARE prediction results showed that the promoter of AcABI5 contains the ABRE, MYB binding sites involved in drought inducibility, and hormone-responsive elements and transcription factor binding sites, among other functional elements (Figure S3). These elements suggest that the expression of AcABI5 may be regulated by abiotic stresses such as salt stress, thereby providing a basis for its involvement in the transcriptional regulation of salt stress responses. The ProtParam analysis showed that AcABI5 has a molecular weight of approximately 40.21 kDa, a theoretical isoelectric point of 9.51, an instability index of 56.46, an aliphatic index of 66.57, and a grand average of hydropathicity of −0.845, indicating that it is hydrophilic (Table S2). SignalP 6.0 predicted no signal peptide, supporting its nuclear localization (Figure S4a). Secondary structure prediction revealed that AcABI5 displays typical structural characteristics of bZIP proteins (Figure S4b), and tertiary structure modeling showed that the protein can form a stable dimer during DNA binding (Figure S4c).

### 2.3. Expression Profile and Subcellular Localization of AcABI5

**Figure 3:**
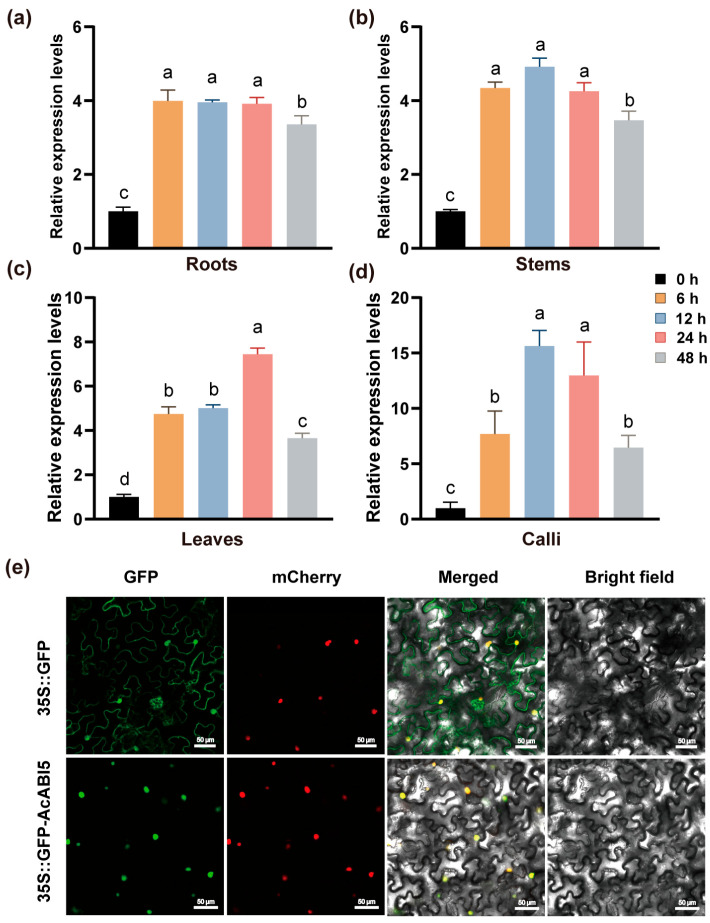
Expression profile and subcellular localization of *AcABI5* under salt stress: (**a**–**d**) Relative expression levels of AcABI5 in roots, stems, leaves, and calli at 0, 6, 12, 24, and 48 h after 200 mM NaCl treatment. Different lowercase letters indicate significant differences by Tukey’s test (means  ±  SD, *n* = 3; *p* < 0.05). (**e**) Subcellular localization of AcABI5 in *Nicotiana benthamiana* leaves. The signals from GFP (green fluorescence), mCherry (red fluorescence), merged (yellow fluorescence), and bright field (no fluorescence) images are shown. The dots in the images represent the nucleus. EGFP: excitation at 488 nm, emission at 507 nm; mCherry: excitation at 587 nm, emission at 610 nm. Scale bars are 50 μm. The experiments were repeated three times with similar results.

To characterize the function of *AcABI5* at the transcriptional and protein levels, we first analyzed its expression profile in different tissues of *A. camelorum* under salt stress, and then determined its subcellular localization. The results showed that *AcABI5* was significantly induced by salt stress in roots, stems, leaves, and calli, and its expression pattern exhibited distinct tissue specificity and temporal dynamics (Figure 3). The roots and stems displayed a rapid response pattern (Figure 3a,b), while the leaves showed a gradual accumulation pattern with expression levels increasing progressively over the course of salt stress (Figure 3c). Calli exhibited the most sensitive and intense response to salt stress, with the relative expression level peaking at 15-fold at 12 h (Figure 3d). These expression patterns suggest that *AcABI5* may be involved in salt stress signal transduction.

As shown in Figure 3e, green fluorescence of 35S::GFP was detected throughout the cells, while green fluorescence was only detected in the nucleus of the cells transfected with the fusion vector 35S::GFP-AcABI5. Moreover, the green fluorescence overlapped well with the red fluorescence of nuclear marker 35S::mCherry-NLS, indicating that the AcABI5 protein localizes to the nucleus and acts as a typical transcription factor.

### 2.4. Transcriptional Activity and Binding Analysis of AcABI5

**Figure 4:**
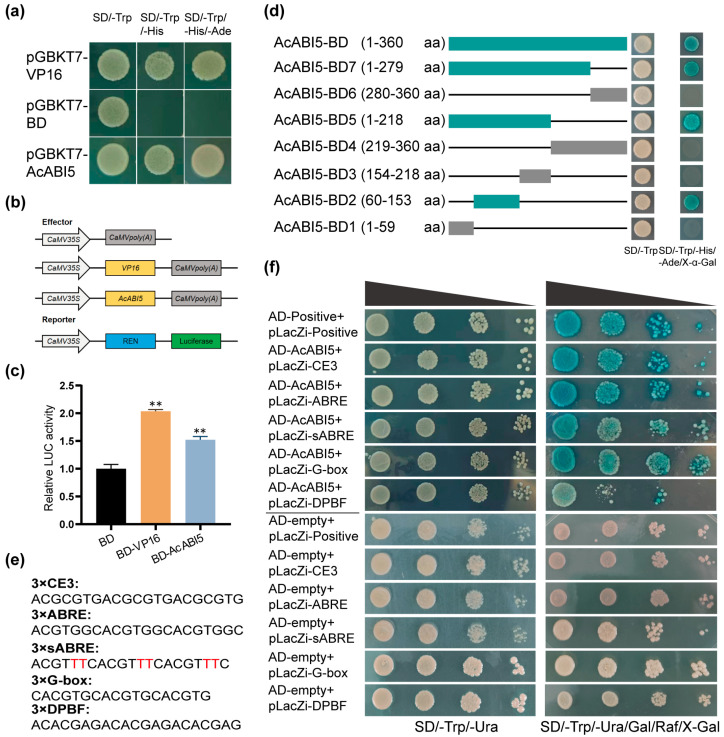
AcABI5 possesses transcriptional activation activity and is able to recognize a variety of core cis-elements in response to ABA: (**a**) Transcriptional activation activity analysis of AcABI5 in yeast cells. VP16 was used as the positive control, and the empty BD vector was used as the negative control. Each construct was tested in at least three independent yeast colonies, and the representative results are shown. (**b**) The carrier structure for effector and reporter groups used in the dual luciferase assay. (**c**) The positive control vector 62-SK-BD-VP16 and the 62-SK-BD-AcABI5 effector were co-infiltrated with the reporter construct into tobacco leaves, respectively. LUC signals were imaged 48 h after infiltration (means  ±  SD, *n* = 3; **, *p* < 0.01; Student’s *t*-test). (**d**) The CDS of *AcABI5* was divided into seven truncated fragments. These seven truncated gene fragments were cloned and constructed into the pGBKT7 vector respectively, and their transcriptional activation activities were verified on the SD/−Trp/−His/−Ade medium containing X-α-Gal. Blue colonies indicate positive transcriptional activation activity. (**e**) Sequences of three tandem repeats of CE3, ABRE, sABRE, G-box, and DPBF elements. (**f**) Y1H analysis of the interaction between AcABI5 and 3 × motifs. AD-Positive + pLacZi-Positive was used as the positive control, and AD-empty was used as the negative control to exclude non-specific background activation. Yeast transformants were adjusted to an OD_600_ of 0.2, serially diluted 10 to 1000 fold, and grown on the SD/−Trp/−Ura and SD/−Trp/−Ura/Gal/Raf/X-Gal media for verification, respectively. Blue colonies indicate that AcABI5 can bind to the corresponding cis-acting element and activate reporter gene expression.

To investigate the transcriptional activity of AcABI5, the CDS was cloned into the pGBKT7 vector. The recombinant plasmid pGBKT7-AcABI5, negative control empty pGBKT7-BD, and positive control pGBKT7-VP16 were separately transformed into Y2HGold yeast competent cells. All three transformant yeast strains grew normally on the SD/−Trp medium (Figure 4a), indicating the successful establishment of the experimental system. Only pGBKT7-AcABI5 and the positive control VP16 could grow on the SD/−Trp/−His/−Ade medium, while the negative control could not grow, indicating that AcABI5 possesses transcriptional activation activity. For further verification, a dual-luciferase assay was performed. The effector vector pGreenII BD-AcABI5 was constructed and co-infiltrated with the reporter vector into *N. benthamiana* leaves (Figure 4b). Compared with the empty BD vector, BD-AcABI5 extremely significantly increased the relative LUC activity, similarly to the positive control BD-VP16 (Figure 4c). These results confirmed that AcABI5 can effectively activate downstream gene transcription in plant cells.

To further identify the core transcriptional activation domain of AcABI5 and clarify the key amino acid region responsible for its transcriptional activation activity, a series of truncated mutants of AcABI5 were constructed and transformed into the yeast strain AH109 for transcriptional activation domain localization. The results showed that AcABI5-BD2 was the shortest truncated mutant with transcriptional activation activity (Figure 4d), suggesting that the transcriptional activation domain of AcABI5 is likely located in the N-terminal region (60–153 aa).

ABI5 has been confirmed to specifically bind to the ABRE located in the promoter regions of target genes in *A. thaliana* [39]. To verify the binding between AcABI5 and ABRE cis-element, a Y1H assay was performed using synthetic sequence constructs. As shown in Figure 4e, each construct harbored three copies of the following cis-elements respectively: ABRE (ACGTGGC); its mutated form sABRE (ACGTTTC); CE3 (ACGCGTG, a key synergistic cis-element for ABRE); G-box (CACGTG, a conserved multifunctional stress-responsive element); and DPBF (ACACGAG, a unique ACACNNG-type sequence that is recognized by ABI5 subfamily transcription factors and lacks the core ACGT motif [40]). The recombinant plasmids pLacZi-motif, pLacZi-positive, pB42AD-AcABI5, and pB42AD-empty were co-transformed into the yeast strain EGY48 as designated experimental and control groups (Figure 4f). All the yeast transformants grew well on the SD/−Trp/−Ura medium. The negative control carrying AD-empty showed only extremely weak background staining on the chromogenic plates, which was markedly lower than the intense blue color observed in the AD-AcABI5 experimental group, thus ruling out non-specific background activation. Among all the experimental groups, only colonies in the DPBF group showed relatively weak growth on the SD/−Trp/−Ura/Gal/Raf/X-Gal chromogenic medium.

Collectively, these results indicate that AcABI5 participates in the transcriptional regulation of target genes under salt stress by binding to these core ABA-responsive cis-elements.

### 2.5. AcABI5 Regulates the Expression of SOD- and POD-Related Genes

Six *A. camelorum* genes highly homologous to *A. thaliana* SOD- and POD-related genes were screened from the DEGs [38], namely *SOD1* (Asp06G031270, Cu/Zn-SOD); *SOD2* (Asp02G005930, Fe-SOD); *SOD3* (Asp05G026070, Mn-SOD); *POD1* (Asp01G018620); *POD2* (Asp08G008140) and *POD3* (Asp07G018810). Promoter cis-acting element prediction revealed that all six genes contain ABRE motifs (Figure S5), indicating that *AcABI5* is highly likely to participate in the regulation of these genes directly or indirectly.

**Figure 5:**
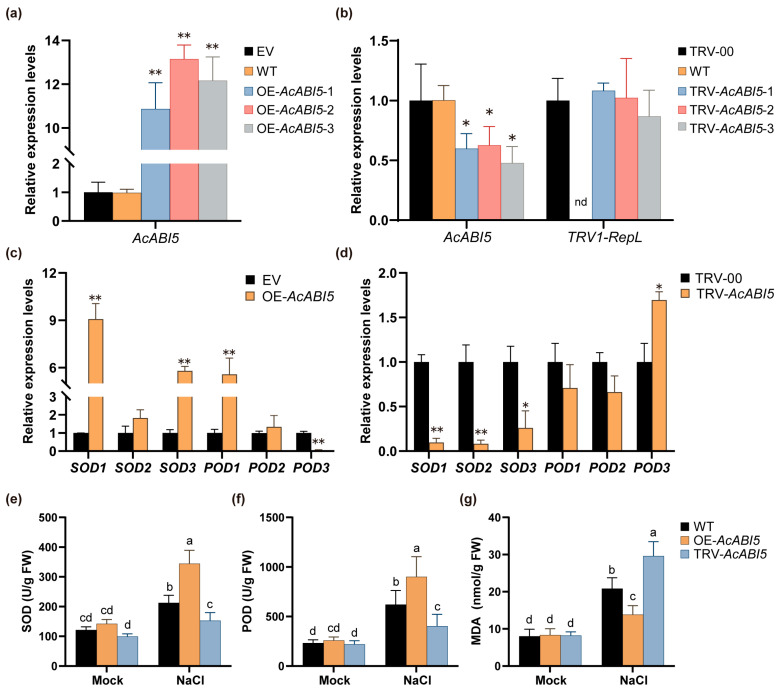
Verification of overexpression and silencing systems and expression analysis of SOD and POD genes under salt stress: (**a**) The *CaMV 35S::AcABI5* overexpression vector was constructed using the pBI121 plasmid, and stably inherited calli were obtained via *A. tumefaciens*-mediated transformation. (**b**) The *AcABI5* gene silencing vector was constructed based on TRV-mediated VIGS, followed by *A.tumefaciens*-mediated infection and transformation. *TRV1-RepL* is the viral replicase large subunit, which indicates whether the pTRV1 vector was successfully introduced into plants and activated. qRT-PCR analysis was performed two weeks after infiltration. nd: not detected. (**c**) Expression analysis of SOD and POD genes in OE-*AcABI5* calli under salt stress for 12 h. (**d**) Expression analysis of SOD and POD genes in TRV-*AcABI5* calli under salt stress for 12 h. (means  ±  SD, *n* = 3; *, *p* < 0.05; **, *p* < 0.01; Student’s *t*-test). (**e**–**g**) Physiological and biochemical indices of *A. camelorum* calli after 12 h of salt stress: SOD activity (**e**), POD activity (**f**), and MDA content (**g**) of WT, OE-*AcABI5*, and TRV-*AcABI5* calli corresponding to panels (**c**,**d**). Different lowercase letters indicate significant differences by Tukey’s test (means  ±  SD, *n* = 3; *p* < 0.05).

To characterize the biological function of *AcABI5*, we constructed *AcABI5* overexpression and tobacco rattle virus (TRV)-mediated virus-induced gene silencing (VIGS) *A. camelorum* calli via *Agrobacterium tumefaciens*-mediated transformation, designated as OE-*AcABI5* and TRV-*AcABI5*, respectively. The transcript levels of *AcABI5* were confirmed by qRT-PCR. In the overexpression system, we identified three independent positive OE-*AcABI5* callus lines with significantly elevated AcABI5 expression, showing a maximum 13-fold increase compared with the empty vector control (EV), whereas no significant difference was observed in the wild type (WT) (Figure 5a). In the silencing system, the transcript level of *AcABI5* was remarkably reduced in three independent TRV-*AcABI5* callus lines relative to the empty vector control (TRV-00) group, and no significant difference was found in the WT (Figure 5b). In addition, *TRV1-RepL* (the viral replicase large subunit, which was amplified to indicate whether the pTRV1 vector had been successfully introduced into the callus and effectively activated) was expressed exclusively in the virus-treated groups, and no target signal was detected in the WT (Figure 5b). These results verified that both the overexpression and silencing vectors were successfully introduced into *A. camelorum* calli and functioned effectively.

Consistent with our physiological data (Figure 5e–g), the molecular analysis revealed that *AcABI5* functions as a positive regulator of the antioxidant defense system in *A. camelorum* calli under salt stress. Overexpression of *AcABI5* significantly upregulated the transcript levels of *SOD1*, *SOD3*, and *POD*1, whereas silencing of *AcABI5* resulted in nearly opposite effects, with significantly reduced expression of *SOD1*, *SOD2*, and *SOD3* (Figure 5c,d). Interestingly, *AcABI5* exhibited a distinct regulatory pattern on *POD3*: in contrast to other POD isoforms, its expression was markedly repressed in OE-*AcABI5* calli but induced in TRV-*AcABI5* calli. Despite the downregulation of *POD3*, the total POD activity in OE-*AcABI5* calli was significantly higher than in WT calli (Figure 5f), indicating that the upregulation of *POD1* and *POD2* is sufficient to drive the overall enhancement of the antioxidant defense capacity. Conversely, the upregulation of *POD3* in TRV-*AcABI5* calli may reflect a stress-induced compensatory response to the compromised expression of other antioxidant genes, although this was insufficient to mitigate the increased reactive oxygen species (ROS) accumulation and subsequent malondialdehyde (MDA) production observed in these lines (Figure 5g).

Collectively, *AcABI5* participates in ROS scavenging under salt stress via the precise modulation of individual antioxidant enzyme genes. These findings provide preliminary insights into the complex regulatory network mediated by *AcABI5* during physiological responses to salt stress.

### 2.6. AcABI5 Confers Salt Tolerance via Regulating ROS Homeostasis in A. camelorum Calli

To further verify the role of *AcABI5* in the salt stress response of *A. camelorum*, we measured the physiological and biochemical indices and performed histochemical staining on WT, OE-*AcABI5*, and TRV-*AcABI5* calli under long-term salt stress in the laboratory.

**Figure 6:**
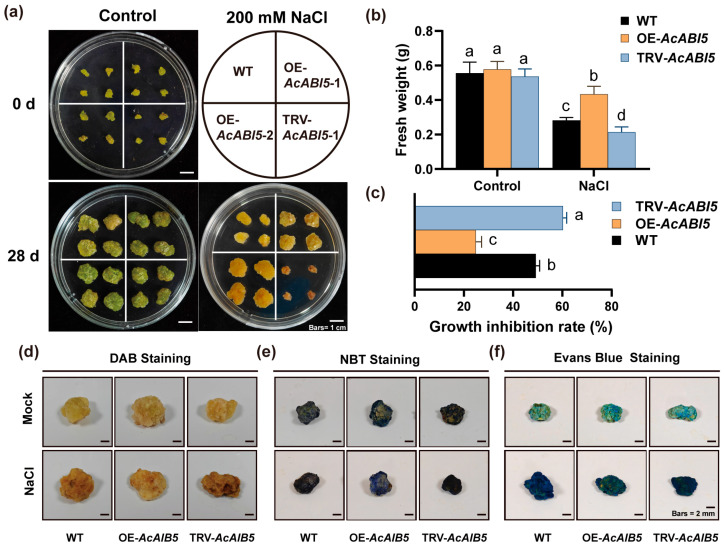
Overexpression and silencing of *AcABI5* reveal its positive role in salt stress tolerance of *A. camelorum* calli: (**a**) Phenotypes of WT, OE-*AcABI5* and TRV-*AcABI5* calli subcultured for 28 d under control and salt stress conditions. Scale bars are 1 cm. (**b**) Fresh weight of WT, OE-*AcABI5* and TRV-*AcABI5* calli grown for 28 d under control and salt stress conditions. (**c**) Growth inhibition rate of WT, OE-*AcABI5* and TRV-*AcABI5* calli under salt stress. Different lowercase letters indicate significant differences by Tukey’s test (means  ±  SD, *n* = 3; *p* < 0.05). (**d**–**f**) WT, OE-*AcABI5,* and TRV-*AcABI5* calli with uniform growth and size were selected and subjected to salt stress for 14 d. (**d**) DAB staining for in situ detection of H_2_O_2_ accumulation, reflecting cellular ROS levels. (**e**) NBT staining for in situ detection of O_2_^−^∙ accumulation, a key ROS in salt stress response. (**f**) Evans blue staining for in situ detection of cell death and membrane integrity. Scale bars are 2 cm. The experiments were repeated three times with similar results.

We observed that under normal culture conditions, WT, OE-*AcABI5*, and TRV-*AcABI5 A. camelorum* calli of the three genotypes exhibited uniform initial status, grew well after 28 days of culture, and showed no significant differences in phenotype or fresh weight, indicating that overexpression or silencing of *AcABI5* did not affect the basal growth of calli, thus eliminating the interference of genotypic basal growth differences on subsequent experiments. After 28 days of exposure to 200 mM NaCl stress, OE-*AcABI5* calli showed only mild growth inhibition, while WT calli had significantly lower volume and fresh weight than OE-*AcABI5* calli; in contrast, TRV-*AcABI5* calli suffered severely inhibited growth, with almost stagnated proliferation, partial tissue browning, and marginal necrosis. Although the fresh weight of OE-*AcABI5* calli also decreased significantly and growth was inhibited under salt stress, they displayed substantially stronger salt tolerance, with a significantly lower growth inhibition rate caused by salt stress than WT and TRV-*AcABI5* calli. Significant differences in fresh weight and growth inhibition rate were observed among the three lines (Figure 6a–c). Conversely, TRV-*AcABI5* calli exhibited markedly higher sensitivity to salt stress and severely impaired salt tolerance. Therefore, overexpression of *AcABI5* significantly enhances the salt tolerance of calli, whereas silencing of *AcABI5* severely impairs their salt tolerance.

Plants rapidly accumulate large amounts of ROS under stress conditions. The main ROS include hydrogen peroxide (H_2_O_2_), hydroxyl radicals (∙OH), and superoxide anion radicals (O_2_^−^∙) [41]. Excessive ROS acts on unsaturated fatty acids in lipids to form peroxides (such as MDA), causing damage to membrane structure and function, oxidizing DNA bases to induce gene mutations or cell apoptosis [42]. POD decomposes H_2_O_2_ to release oxygen ions, which oxidize DAB to form brown water-insoluble precipitates. The darker the precipitate color, the higher the H_2_O_2_ content in calli and the more severe the damage. O_2_^−^∙, one of the ROS, can reduce NBT to form blue deposits, thus enabling localization of O_2_^−^∙ production sites in calli. The darker the color, the higher the ROS content and the more severe the cell damage. Further histochemical staining results showed that, compared with WT and TRV-AcABI5 calli, O_2_^−^∙ in OE-*AcABI5* calli accumulated primarily on the side in contact with the culture medium, with markedly reduced O_2_^−^∙ levels at the callus tips distant from the medium. Overexpression of *AcABI5* reduced the accumulation of ROS and MDA and alleviated cell death, whereas silencing of *AcABI5* led to excessive ROS accumulation and aggravated cell death (Figure 6d–f). Taken together, these results suggest that *AcABI5* acts as a positive regulator of the salt stress response in *A. camelorum* calli. Overexpression of *AcABI5* significantly enhanced the activities of antioxidant enzymes such as SOD and POD, thereby effectively scavenging ROS, reducing membrane lipid peroxidation, and ultimately decreasing cell death to improve the tolerance of calli to salt stress.

### 2.7. VIGS-Mediated Silencing of AcABI5 Increases Salt Sensitivity in N. benthamiana

**Figure 7:**
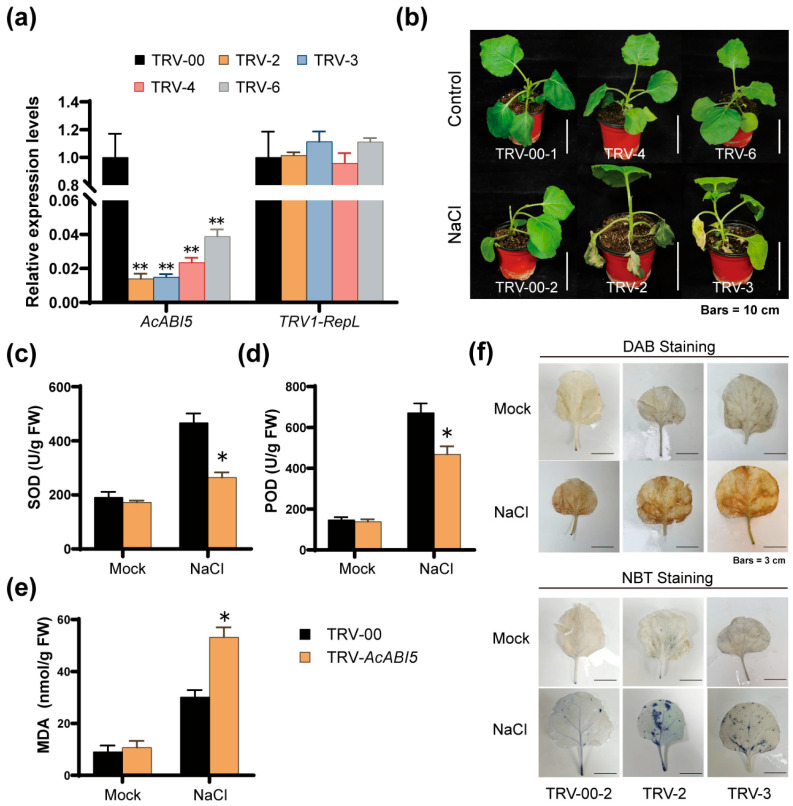
Silencing *AcABI5* reduces the salt tolerance of *N. benthamiana*. WT *N. benthamiana* plants with vigorous growth were selected for *A. tumefaciens* infiltration, with pTRV1 + pTRV2-AcABI5 as the experimental group and pTRV1 + pTRV-00 as the empty vector control group: (**a**) Expression levels of *AcABI5* and *TRV1-RepL* in empty vector control (TRV-00) and *AcABI5*-silenced (TRV-*AcABI5*: lines 2, 3, 4, and 6) *N. benthamiana* plants under normal culture conditions at three weeks after VIGS infiltration. *TRV1-RepL* is the viral replicase large subunit, which indicates whether the pTRV1 vector was successfully introduced into plants and activated. *NbEF1α* was used as the reference gene (means  ±  SD, *n* = 3; **, *p* < 0.01; Student’s *t*-test). (**b**) After two weeks of salt stress treatment, the TRV-*AcABI5 N. benthamiana* plants exhibited a stronger salt-sensitive phenotype than TRV-00 plants. Scale bars are 10 cm. (**c**–**e**) SOD activity (**c**), POD activity (**d**), and MDA content (**e**) in TRV-00 and TRV-*AcABI5 N.benthamiana* plants after two weeks of salt stress (means  ±  SD, *n* = 3; *, *p* < 0.05; Student’s *t*-test). (**f**) DAB staining of *N. benthamiana* leaves for in situ detection of H_2_O_2_ accumulation, reflecting cellular ROS levels (**upper panel**); NBT staining for in situ detection of O_2_^−^∙ accumulation, a key ROS in the salt stress response (**lower panel**). Scale bars are 3 cm. The experiments were repeated three times with similar results.

To further elucidate the response mechanism of *AcABI5* to salt stress, *AcABI5* was heterologously silenced in the model plant *N. benthamiana* using VIGS. Three weeks after *A. tumefaciens* infiltration, the transcript level of *AcABI5* was significantly reduced, verifying the normal function of the VIGS system. Compared with the TRV-00 control, *AcABI5* was efficiently silenced in four independent *N. benthamiana* lines (TRV-*AcABI5*: TRV-2, TRV-3, TRV-4, and TRV-6) (Figure 7a). Plants of the TRV-*AcABI5* lines and the empty vector control lines (TRV-00: TRV-00-1, TRV-00-2) were treated with salt stress for two weeks, with deionized water treatment as the control group. Under normal growth conditions, the TRV-00 and TRV-*AcABI5* plants showed similar growth vigor with no obvious phenotypic differences. After salt stress treatment, leaves of TRV-*AcABI5 N. benthamiana* exhibited severe wilting, drooping, browning, and necrosis, whereas the TRV-00 plants only displayed slight leaf drooping and maintained better overall growth status (Figure 7b).

Meanwhile, the accumulation level of ROS was evaluated by measuring the activities of SOD and POD, the content of MDA, and histochemical staining. The results showed that the activities of SOD and POD were both upregulated in the TRV-00 and TRV-*AcABI5* plants under salt stress, but the enzymatic activities in the TRV-*AcABI5* lines were significantly lower than those in the TRV-00 lines, accompanied by remarkably higher MDA accumulation (Figure 7c–e). Histochemical staining showed only a small number of brown and blue deposits on leaves of all the plants under mock treatment, while the staining intensity of DAB and NBT deposits on leaves was markedly enhanced under NaCl treatment. Comparison between the TRV-00 and TRV-*AcABI5* plants revealed much darker staining in TRV-*AcABI5* leaves (Figure 7f), indicating that silencing of *AcABI5* led to excessive ROS accumulation in *N. benthamiana* leaves under salt stress.

Combined with phenotypic observations and physiological and enzymatic index data, we conclude that silencing of *AcABI5* inhibits the function of the antioxidant enzyme system, reduces the ROS-scavenging capacity of *N. benthamiana*, and thus renders *N. benthamiana* more sensitive to salt stress.

## 3. Discussion

Soil salinization has long posed a severe threat to the sustainable development of agriculture. Faced with harsh environments, plants cannot migrate like animals and can only passively adapt to biotic or abiotic stresses. *A. camelorum*, as a dominant leguminous shrub in the Taklamakan Desert, exhibits extremely strong adaptability to high-salt and arid conditions, making it an ideal model for studying the salt tolerance mechanisms of desert plants. However, the molecular regulatory basis of its salt tolerance remains unclear. Studies have shown that the bZIP transcription factor family, especially the ABI5 subfamily, is extensively involved in the regulation of plant gene expression under abiotic stresses by mediating the ABA signaling pathway and regulating the antioxidant system. Although homologous *ABI5* genes have been identified in species such as *A. thaliana*, wheat, and soybean, the function and regulatory mechanism of *ABI5* in *A. camelorum* have not been reported. Therefore, we cloned, characterized, and functionally validated a novel gene, *AcABI5*, which encodes a bZIP transcription factor and plays a role in enhancing salt tolerance.

The bioinformatics and subcellular localization analyses confirmed that *AcABI5* encodes a typical nuclear-localized bZIP transcription factor, containing a conserved N-X_7_-R/K basic region and an L-X_6_-L leucine zipper domain. The prediction results showed that the *AcABI5* promoter contains ABRE and MYB drought-responsive elements (Figure S3), which explains why salt stress can significantly induce *AcABI5* expression. This indicates that its expression itself is regulated by salt stress signals, serving as a key node in the salt stress response pathway of *A. camelorum*, consistent with the known function of *ABI5* as a core component of ABA-mediated stress responses [26,31].

Transcriptional activation activity is a core functional characteristic of transcription factors. Y2H and dual-luciferase assays confirmed that AcABI5 exhibits strong transcriptional activation activity in both yeast and plant cells (Figure 4a,c), which is consistent with the functional conservation of ABI5 homologous proteins in chickpea and lentil [43,44]. The truncation analysis further localized the core transcriptional activation domain of AcABI5 to the N-terminal 60–153 amino acid region (Figure 4d), providing a target for future studies on its post-translational modifications and protein–protein interactions [45].

It is well established that ABI5 regulates transcription by specifically binding to the ABRE in the promoter regions of target genes [39]. Y1H assays in this study showed that AcABI5 binds to ABRE, consistent with Utsugi et al.’s finding that TaABI5 activates the promoter of Em containing ABRE [46]. AcABI5 also binds to CE3 (a synergistic element of ABRE) and G-box (a multifunctional stress response element). DPBF lacks the core ACGT motif [40], and AcABI5 exhibits weak binding affinity to it (Figure 4e,f), indicating a certain degree of specificity in its DNA binding. This reflects the adaptive adjustment of *A. camelorum* to desert environments during evolution. Furthermore, as previously mentioned, certain transcription factors also interact with each other to jointly regulate downstream gene expression. For example, *Arabidopsis* ECT8 is regulated by the ABI5/ABF-mediated ABA signaling pathway in response to ABA and abiotic stress [47]. Collectively, these results confirm that AcABI5, as a transcriptional activator in the ABA signaling pathway, mediates salt stress responses by recognizing multiple ABA-related cis-elements.

The expression pattern of a gene is usually closely related to its function. The qRT-PCR results showed that *AcABI5* is significantly induced by salt stress in the roots, stems, leaves, and calli of *A. camelorum*, but exhibits distinct temporal dynamic characteristics (Figure 3). Roots and stems showed a rapid induction pattern, suggesting that *AcABI5* may be involved in early signal transduction in these salt stress-perceiving organs; *AcABI5* expression in leaves showed a gradual accumulation trend, implying its role in long-term salt stress adaptation. Notably, the response of *AcABI5* in calli was the most intense, with a 15-fold upregulation at 12 h (Figure 3d), which is consistent with the strong salt tolerance phenotype observed in OE-*AcABI5* calli. *OsABI5-Like1* is specifically expressed in various tissues of rice and regulates abscisic acid and auxin [48]. The tissue-specific expression pattern indicates that *AcABI5* plays a comprehensive regulatory role in the salt stress response of different organs of *A. camelorum*, highlighting its core position in the plant’s salt tolerance regulatory network.

Salt stress induces excessive accumulation of ROS, which causes oxidative damage to cell membranes and biomacromolecules [49,50]. The antioxidant system, composed of enzymes such as SOD and POD, is crucial for plants to scavenge ROS and resist salt stress [15]. This study confirmed that AcABI5 acts as a positive regulator of the salt stress response in *A. camelorum* calli: overexpression of *AcABI5* significantly increased SOD and POD activities, reduced ROS accumulation and MDA content, and alleviated growth inhibition caused by salt stress; in contrast, silencing *AcABI5* showed the opposite phenotype (Figure 5e–g and Figure 6). These results are consistent with previous studies reporting that ABI5 transcription factors enhance salt tolerance by activating the antioxidant system [51,52]. Mechanistically, AcABI5 precisely regulates the expression of multiple SOD and POD isoform genes (Figure 5c,d). Overexpression of *AcABI5* upregulated the transcriptional levels of *SOD1* (Cu/Zn-SOD), *SOD3* (Mn-SOD), and *POD1*, while silencing *AcABI5* significantly downregulated the expression of *SOD1*, *SOD2* (Fe-SOD), and *SOD3*. Notably, AcABI5 exhibited a unique regulatory pattern on *POD3*: its expression was repressed in overexpression lines but induced in silenced lines. This differential regulation suggests a complex fine-tuning mechanism of AcABI5 in the antioxidant system. The upregulation of *POD1* and *POD2* in overexpression lines was sufficient to enhance total POD activity, indicating functional redundancy among POD isoforms; the induction of *POD3* in silenced lines may be a stress-induced compensatory response, but due to the downregulation of key SOD and POD genes, this compensation failed to alleviate ROS accumulation. The promoter analysis showed that all six SOD and POD genes contain ABRE motifs (Figure S5), and combined with the binding ability of AcABI5 to ABRE in Y1H assays, it is speculated that AcABI5 may directly bind to the promoter regions of these genes to regulate their transcription. In summary, this study initially established a regulatory pathway: salt stress induces *AcABI5* expression, AcABI5 binds to the ABREs in the promoter regions of SOD and POD genes, regulates their expression levels, enhances antioxidant enzyme activities, scavenges excessive ROS, reduces membrane lipid peroxidation, and ultimately improves the salt tolerance of *A. camelorum*. Song et al. performed a comprehensive study on *MaABI5-like* in banana and found that VIGS-mediated silencing of *MaABI5-like* in fruit inhibited the expression of downstream ABA-related stress-responsive genes, aggravated membrane damage and exacerbated chilling injury, thus verifying that *MaABI5-like* positively regulates cold tolerance [53]. To further confirm the regulatory mechanism of *AcABI5* in response to salt stress, we heterologously silenced *AcABI5* in *N. benthamiana* and obtained results similar to those observed in *A. camelorum* calli. *N. benthamiana* with silenced *AcABI5* exhibited reduced salt tolerance under salt stress, displaying more severe wilting and necrosis compared with the empty vector control lines, and their ROS scavenging capacity was also significantly decreased (Figure 7). Li et al. demonstrated that overexpression of *NtbZIP62* in *N. benthamiana* significantly enhanced salt stress tolerance in transgenic plants [54].

In conclusion, this study is the first to clone and identify the bZIP transcription factor gene *AcABI5* from *A. camelorum*. It confirms that AcABI5 is a nuclear-localized protein with transcriptional activation activity, which can bind to ABA-responsive cis-elements and regulate the expression of SOD and POD antioxidant enzyme genes, filling the research gap in the salt tolerance mechanism of *A. camelorum*. In addition, an efficient *A. camelorum* callus experimental system was established, providing technical support for functional genomics research on this non-model desert plant (Figure S1, Table S1). At the application level, *AcABI5* is a potential candidate gene for the genetic improvement of salt-tolerant crops. Given the evolutionary relevance between *A. camelorum* and important leguminous crops such as *C. arietinum* (Figure 2b), heterologous expression of *AcABI5* may enhance the salt tolerance of these crops, providing support for the sustainable development of agriculture in saline–alkaline areas. Furthermore, the identification of *AcABI5* enriches the gene resource pool for saline–alkaline land remediation and provides a basis for the development of eco-friendly strategies for desertification control.

Despite the above progress, this study still has certain limitations: First, due to the long time required for genetic transformation, functional characterization was mainly performed using the callus system, and the salt tolerance function of *AcABI5* in the whole *A. camelorum* plants have not been confirmed. Second, the upstream regulators of *AcABI5* and its interacting proteins in the ABA signaling pathway have not been identified, and the complete regulatory network remains unclear. Third, the mechanistic evidence supporting the function of AcABI5 is still largely correlative and needs to be further explored in depth.

Future research should address these limitations by: (1) constructing *AcABI5* overexpression and silencing transgenic *A. camelorum* plants to verify its salt tolerance function at the whole-plant level; simultaneously performing heterologous expression in *Arabidopsis* or soybean to evaluate its crop improvement potential; (2) verifying the direct binding of AcABI5 to the promoters of *SOD* and *POD* genes by EMSA and ChIP-qPCR assays, and screening interacting proteins and upstream regulatory factors of AcABI5 using Y2H, Co-IP and other techniques to clarify the complete salt stress regulatory pathway of *AcABI5*; (3) conducting rescue experiments with exogenous ROS scavengers on WT and *AcABI5*-silenced calli under salt stress, so as to verify whether ROS homeostasis is the key pathway responsible for *AcABI5*-mediated salt tolerance.

## 4. Materials and Methods

### 4.1. Plant Material and Callus Induction

The *A. camelorum* seeds used in this study were collected in July 2023 from the Cele Desert Grassland Ecosystem National Field Scientific Observation and Research Station, Xinjiang Institute of Ecology and Geography, Chinese Academy of Sciences. Mature and plump seeds were vernalized for 48 h, soaked in 75% ethanol for 30 s, and rinsed 3 times with sterile distilled water, followed by immersion in 5% NaOCl for 5 min and washed 5 times with sterile distilled water. The seeds were inoculated on MS medium [55] supplemented with 3% (*w*/*v*) sucrose and 0.3% (*w*/*v*) phytagel (pH = 5.8–6.0), and grown under conditions of 25 ± 1 °C, 3000 lx, 16 h light and 8 h dark.

We selected aseptic seedlings that were in good growth condition and had a seedling age of approximately 28 days for the callus culture of *A. camelorum*. Young stem segments of approximately 0.5 cm were cut as explants and transferred to culture media containing different concentration combinations of 2,4-dichlorophenoxyacetic acid (2,4-D) and 6-benzylaminopurine (6-BA) for callus induction. The culture conditions were the same as those described previously.

### 4.2. Determination of Salt Stress Concentration

To determine the optimal salt stress concentration, the *A. camelorum* seeds were inoculated onto salt-stress MS medium supplemented with NaCl at gradient concentrations of 0 mM, 50 mM, 100 mM, 150 mM, 200 mM, 250 mM, 300 mM, and 400 mM, respectively [2]. The treatment protocol was consistent with that described in Section 4.1 of this study. For each treatment, 50 seeds were inoculated per flask, with three biological replicates set up in parallel. The germination rate, length of shoots, main root length and overall growth status of the *A. camelorum* seedlings were observed and recorded within 21 days.

### 4.3. RNA Extraction, cDNA Preparation, and qRT-PCR Analysis

Total RNA was extracted from the samples according to the instructions of the TIANGEN Total RNA Extraction Kit (DP441, TIANGEN, Beijing, China). The concentration and purity of RNA were determined using a Nanodrop (Thermo Fisher Scientific, Waltham, MA, USA), and the integrity of RNA was detected by agarose gel electrophoresis. RNA was reverse-transcribed into cDNA as a template using the TIANGEN FastKing One-Step Genomic DNA Removal and cDNA Synthesis SuperMix according to the instructions [56].

cDNAs from the control and treatment groups were used as the template. qRT-PCR was performed according to the instructions of Vazyme SYBR qRT-PCR Master Mix (Q712-02, Vazyme, Nanjing, China). *A. camelorum EF-1α* (Asp06G015730) and *ACT* (Asp07G013640) were used as internal reference genes. qRT–PCR was performed using Applied Biosystems QuantStudio 1 Real-Time PCR System (Thermo Fisher Scientific, Foster City, CA, USA), and the 2^−ΔΔCt^ method was used to calculate the expression levels of each gene in *A. camelorum* [57]. All the experiments were replicated at least three times. The primers used for qRT–qPCR are listed in Table S2.

### 4.4. Cloning and Sequence Analysis of AcABI5

*A. camelorum* leaves, shoots and calli cDNA were used as templates to amplify the full-length CDS of *AcABI5* (Asp07G015930) using gene-specific primers (Table S2).

The methods used for the bioinformatics analysis are referenced from previous studies [58,59]. The Expasy-ProtParam tool (https://web.expasy.org/protparam/, accessed on 1 May 2025) was used to analyze the physicochemical properties of the predicted Asp07G015930 protein. SignalP 6.0 (https://services.healthtech.dtu.dk/services/SignalP-6.0/, accessed on 1 May 2025) was used to predict the signal peptide of the full-length sequence to determine its subcellular localization characteristics. Homologous sequences of Asp07G015930 were obtained using NCBI Blast (https://blast.ncbi.nlm.nih.gov/Blast.cgi/, accessed on 1 May 2025) and Uniprot (https://www.uniprot.org/, accessed on 1 May 2025), and 38 homologous sequences were screened for subsequent analysis. Multiple sequence alignment was performed using the ClustalW algorithm implemented in MEGA 11 software, and conserved domains were retained for phylogenetic tree construction. The phylogenetic tree was constructed using the Neighbor-Joining (NJ) method in MEGA 11, with the Jones–Taylor–Thornton (JTT) amino acid substitution model. Branch support was evaluated with 1000 bootstrap replicates, and the tree was rooted at the midpoint to standardize the topology. After construction, the generated phylogenetic tree was optimized and visualized using the online tool ITOL (http://itol.embl.de/, accessed on 3 May 2025). The protein sequences were submitted to the NCBI-CDD (https://www.ncbi.nlm.nih.gov/cdd/, accessed on 3 May 2025) to predict conserved domains and identify the bZIP superfamily domain and other related functional domains. The online tool MEME (https://meme-suite.org/meme/tools/meme, accessed on 3 May 2025) was used for the conserved motif analysis, with 10 motifs set and a motif length range of 6–50 amino acids. Cis-acting regulatory elements of the promoter were predicted using PlantCARE (https://bioinformatics.psb.ugent.be/webtools/plantcare/html/, accessed on 3 May 2025). The TBtools-II software (version 2.441)was used to visualize conserved motifs and generate domain and motif band diagrams [60]. The online tool SOPMA program (https://npsa-prabi.ibcp.fr/cgi-bin/npsa_automat.pl?page=/NPSA/npsa_server.html/, accessed on 1 May 2025) was used to predict the secondary structure of the protein. The online tool SWISS MODEL (https://swissmodel.expasy.org/, accessed on 1 May 2025) was used to predict the tertiary structure of the protein, generate a 3D structure model based on homology modeling, and evaluate the structure quality.

### 4.5. Subcellular Localization

To examine the subcellular localization of AcABI5 using SnapGene 6.0.2, specific primers for the *AcABI5* gene were designed based on its CDS sequence, incorporating attB-specific recombination sites of the Invitrogen Gateway^®^ donor vector pDONR-Zeo (Cat. No. 12535035, Invitrogen, Carlsbad, CA, USA) (Table S3) [61]. Using pDONR-Zeo as the donor vector, the entry vector was obtained through BP reaction according to the instructions of Invitrogen Gateway™ BP Clonase™ II Enzyme Mix (Cat. No. 11789020, Invitrogen, Carlsbad, CA, USA). Using pK7WGF2, a recipient vector containing the enhanced green fluorescent protein (eGFP) reporter gene as the recipient vector, the expression vector was obtained through LR reaction according to the instructions of Invitrogen Gateway^®^ LR Clonase™ II Enzyme Mix (Cat. No. 11791020, Invitrogen, Carlsbad, CA, USA). The constructed pK7WGF2-35S::GFP-AcABI5 vector plasmid was transformed into *A*. *tumefaciens* GV3101. The empty GFP vector was used as a control, and the mCherry-NLS (a nuclear-localized mCherry fusion with a classic nuclear localization signal) vector was used as a marker for co-injection into *N*. *benthamiana* leaves. Observation was performed using a laser confocal microscope after 48 h. Fluorescence was detected with a Zeiss LSM 800 (Carl Zeiss AG, Oberkochen, Germany) confocal microscope [62]. The primers used for vector construction and identification are listed in Table S3.

### 4.6. Transcriptional Activity Assay

The transcriptional activation activity of AcABI5 was detected using a Yeast Transcriptional Activation Activity Detection Kit (MH101, Coolaber, Beijing, China) [63]. The CDS sequence of *AcABI5* was constructed into the pGBKT7 vector by homologous recombination, with VP16 as the positive control and the empty pGBKT7 vector as the negative control. The resulting recombinant plasmids were transformed into yeast strain Y2HGold and cultured on the SD/−Trp deficient medium at 30 °C for 3 d. The transformants were then transferred to the SD/−Trp/−Ade/−His triple-deficient screening medium.

The design of the dual-luciferase assay was based on a previous report [64], and the detection was carried out using a Plant Transcriptional Activity Detection Kit (FZ1035, PYEAST, Wuhan, China). The CDS sequence of AcABI5 was inserted into the multiple cloning site (MCS) downstream of the BD domain in the pGreenII62-SK-BD vector by homologous recombination. The empty pGreenII62-SK-BD vector was used as the negative control, and pGreenII62-SK-BD-VP16 was used as the positive control. The recombinant vectors were transformed into *A. tumefaciens* strain GV3101 harboring the pSoup helper plasmid, and then injected into tobacco leaves for infiltration. At 48 h post-infiltration, the infiltrated leaves were cut into pieces by region and immediately transferred into liquid nitrogen, ground thoroughly into a fine powder, and the luciferase activity was subsequently determined following the manufacturer’s instructions. LUC and REN activities were assayed; using REN as the internal reference, the LUC/REN ratio was calculated to reflect the relative promoter activity. The primers used for vector construction and identification are listed in Table S3.

### 4.7. Transcriptional Activation Domain Verification

Using the Yeast AH109-GAL4 Two-Hybrid interaction proving kit (YH2021, Coolaber, Beijing, China), the CDS of *AcABI5* was divided into seven truncated fragments. These seven truncated gene fragments were cloned and constructed into the pGBKT7 vector respectively, and their transcriptional activation activities were verified on the SD/−Trp/−His/−Ade medium containing X-α-Gal. All the experiments were replicated at least three times. The primers used for vector construction and identification are listed in Table S3.

### 4.8. Yeast One-Hybrid Assay

Using EGY48-LacZ Yeast One-Hybrid Interaction Verification Kit (YH3010-10T, Coolaber, Beijing, China). The CDS sequence of AcABI5 was inserted into the MCS downstream of the AD domain in the pB42AD vector by homologous recombination. Complementary oligos were designed and annealed to generate three tandem motif fragments with *EcoR*I and *Xho*I sticky ends, which were then inserted into the respective pLacZi vectors using T4 DNA ligase. Each construct harbored three copies of the following cis-elements respectively: ABRE (ACGTGGC), sABRE (ACGTTTC), CE3 (ACGCGTG), G-box (CACGTG), and DPBF (ACACGAG) [64]. The recombinant plasmid pLacZi-motif and pB42AD-AcABI5 were co-transformed into the yeast strain EGY48. Then, the cells were cultured on the SD/−Trp/−Ura and SD/−Trp/−Ura/Gal/Raf/X-Gal plates at 30 °C for 3–4 d. The growth status and blue color development were observed. The primers used for vector construction and identification are listed in Table S3.

### 4.9. Determination of Antioxidant Enzyme Activity and MDA Content

The activity of SOD and POD, and contents of MDA in the calli of *A. camelorum* were determined in three biological replicates, using the MDA Kit (FXs0584), SOD Kit (FXs0566) and POD Kit (FXs0465) following the manufacturer’s instructions (Shanghai Fenxi Biotechnology Co., Ltd., Shanghai, China). Detailed instructions are available at https://www.shfxsw.cn (accessed on 23 June 2025).

### 4.10. Generation of Overexpressing and VIGS-Silenced Calli and N. benthamiana Plants

The TRV-mediated VIGS vectors pTRV1 and pTRV2 were used to silence the *AcABI5* gene. The CDS sequence of *AcABI5* was constructed into the pBI121 vector via homologous recombination to generate the *AcABI5* overexpression vector. A 300 bp specific fragment was designed from the CDS while avoiding the conserved domain; this fragment was cloned and inserted into the pTRV2 vector by homologous recombination to construct the silencing vector [59]. The empty pBI121 vector, pBI121-35S::AcABI5, pTRV1, empty pTRV2 vector, and pTRV2-AcABI5 were first transformed into *Escherichia coli* DH5α competent cells and incubated on LB agar plates with the corresponding antibiotics at 37 °C. Single colonies with correct sequencing were selected for plasmid extraction, and the extracted plasmids were subsequently transformed into *A. tumefaciens* GV3101 competent cells. The transformed *A. tumefaciens* was cultured on selective medium containing 25 mg/L rifampicin and vector-specific antibiotics for 42–72 h. *A. tumefaciens* single colonies with verified sequencing were subjected to expansion culture, then resuspended in an infection solution containing 100 μM acetosyringone (AS), and statically incubated for 3 h for subsequent callus infection. The empty pBI121 vector served as the EV control, and the empty pTRV2 vector was used as the TRV-00 control. Co-transformation of calli and *N. benthamiana* leaves was achieved via co-infection with pTRV1 and pTRV2. The primers used for vector construction and identification are listed in Table S3.

### 4.11. Histochemical Staining

The histochemical staining method was improved on the basis of previous work [65,66,67]. *A. camelorum* calli with uniform size were placed in 50 mL centrifuge tubes. Prepared DAB staining solution, NBT staining solution and Evans blue solution were added, respectively, to submerge the calli. Vacuum was slowly applied to 0.8 MPa and maintained for 5 min, then slowly restored to normal pressure. Staining was performed at room temperature in the dark for 6–12 h. After discarding the staining solution, the calli were rinsed repeatedly with sterile distilled water to remove residual staining solution. Decolorizing solution was added, and the samples were incubated in a 95 °C water bath for 10–30 min until chlorophyll was completely removed.

DAB solution (1 mg/mL): Weigh out 100 mg of DAB powder and dissolve it in 100 mL of 0.1 mol/L phosphate-buffered solution (pH = 7.4) to prepare a 1 mg/mL aqueous solution. Adjust the pH to 3.8 with hydrochloric acid, and store the solution at 4 °C in the dark.

NBT solution (0.5 mg/mL): Weigh out 50 mg of NBT powder and dissolve it in 100 mL of 0.1 mol/L phosphate-buffered solution (pH = 7.4). Store the solution at 4 °C in the dark.

Evans blue solution (0.5 mg/mL): Weigh out 50 mg of Evans blue powder and dissolve it in 100 mL of double-distilled water. After dissolving thoroughly, store the solution at room temperature in the dark.

Decolorizing solution: Mix 95% ethanol, acetic acid, and glycerol at a volume ratio of 3:1:1 and store at room temperature in the dark.

### 4.12. Statistical Analysis

Statistical analysis and figure plotting were performed using the GraphPad Prism software (Version 10.1.2, GraphPad Software Inc., San Diego, CA, USA). Student’s *t*-test was used for comparisons between two groups, and one-way ANOVA was employed for comparisons among three or more groups. All the data were calculated from at least three independent biological replicates for each treatment. Different lowercase letters indicate significant differences by Tukey’s test (*p* < 0.05). Asterisks are used to indicate significant differences in Student’s *t*-test (*, *p* < 0.05; **, *p* < 0.01). All the results are presented as means ± standard deviation (SD). Error bars in the figures indicate SD.

## 5. Conclusions

AcABI5 from *A. camelorum* belongs to the bZIP transcription factor family, and it enhances the salt tolerance of *A. camelorum* by regulating the antioxidant defense system, including SOD and POD genes. The *AcABI5* gene identified in this study enriches the genetic resource pool for salt tolerance research and provides new insights into the adaptive strategies of desert plants under extreme saline–alkaline stress.

## Data Availability

Data from this study will be made available upon reasonable request.

## Supplementary Materials

The following supporting information can be downloaded at https://www.mdpi.com/article/10.3390/ijms27093812/s1.

## Abbreviations

The following abbreviations are used in this manuscript:

2,4-D	2,4-dichlorophenoxyacetic acid
6-BA	6-benzylaminopurine
ABA	Abscisic acid
ABI5	Abscisic acid-insensitive 5
ABRE	Abscisic acid-responsive element
Ade	Adenine
AS	Acetosyringone
bZIP	The basic leucine zipper
ChIP-qPCR	Chromatin immunoprecipitation-quantitative PCR
Co-IP	Co-immunoprecipitation
DAB	3,3′-diaminobenzidine
DEGs	Differentially expressed genes
EMSA	Electrophoretic mobility shift assay
EV	Empty vector
Gal	Galactose
His	Histidine
Leu	Leucine
MDA	Malondialdehyde
MES	2-morpholinoethanesulfonic acid
NBT	Nitro blue tetrazolium
PCR	Polymerase chain reaction
POD	Peroxidase
qRT-PCR	Quantitative real-time PCR
Raf	Raffinose
Rif	Rifampicin
ROS	Reactive oxygen species
SOD	Superoxide dismutase
Trp	Tryptophan
TRV	Tobacco rattle virus
Ura	Uracil
VIGS	Virus-induced gene silencing
WT	Wild type
Y1H	Yeast one-hybrid assay
Y2H	Yeast two-hybrid assay
