# Supplementary material for: De Novo Functional Characterization of AcABI5 Transcription Factor and Its Role in Physiological Responses to Salt Stress in Alhagi camelorum Callus
Source: Int J Mol Sci. 2026 Apr 24;27(9):3812. doi: 10.3390/ijms27093812 (PMC13163759; doi:10.3390/ijms27093812)
Supplement: Supplementary file 1 [file ijms-27-03812-s001.zip › Supplementary Information 1.pdf]

Supplementary Information

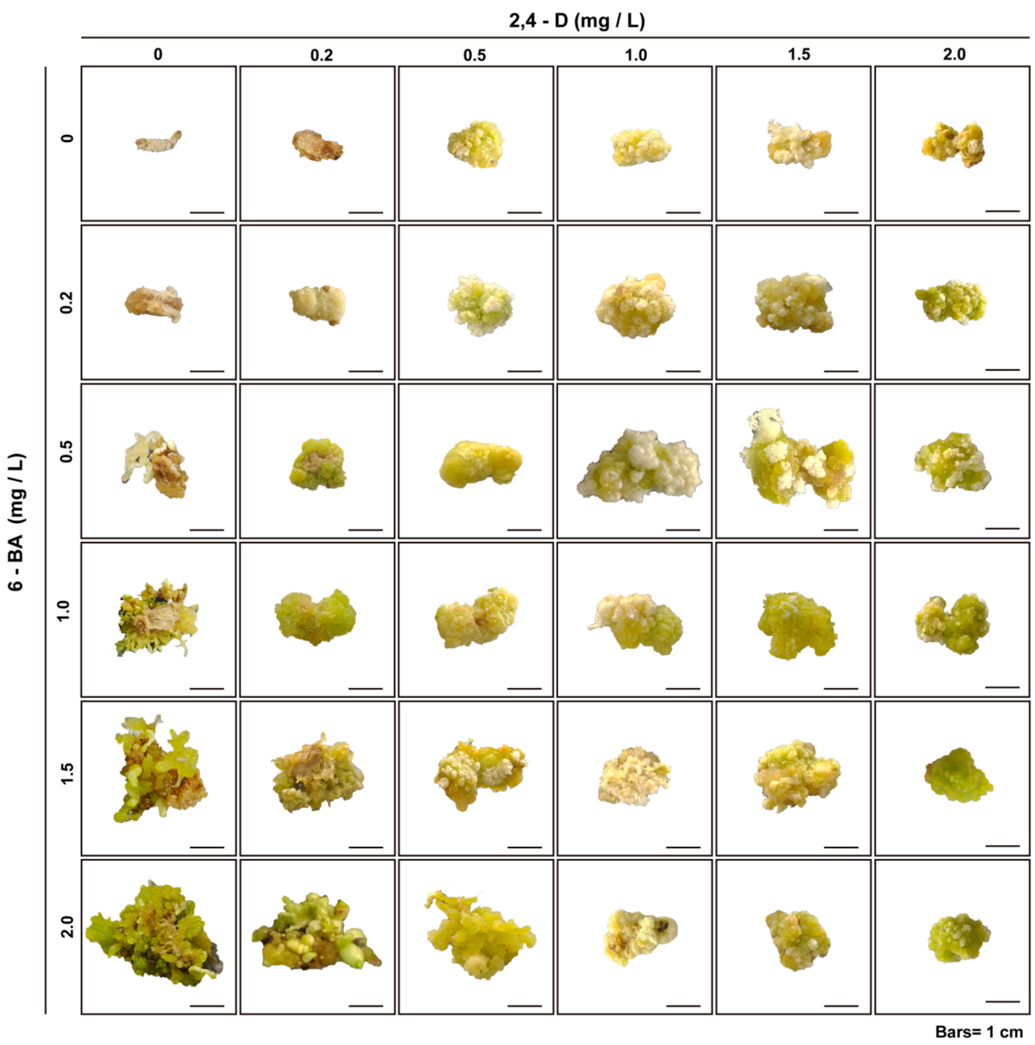

Figure S1. Effects of different hormone concentrations on callus induction.The abscissa represents the concentration of 2,4-D (0–2 mg/L), the ordinate represents the concentration of 6-BA (0–2 mg/L), and each grid corresponds to one type of hormone combination. Scale Bars are 1 cm.

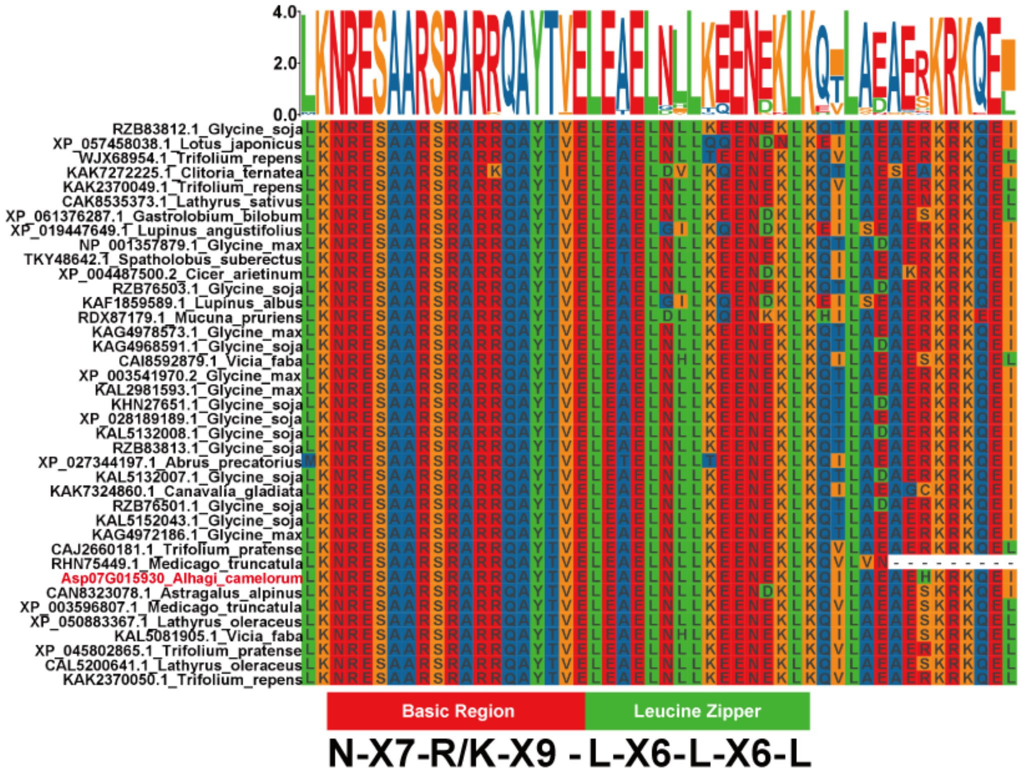

Figure S2. Multiple sequence alignment and conserved motifs analysis revealed that the ABI5 domain of *A. camelorum* consists of a basic region and a leucine zipper region.

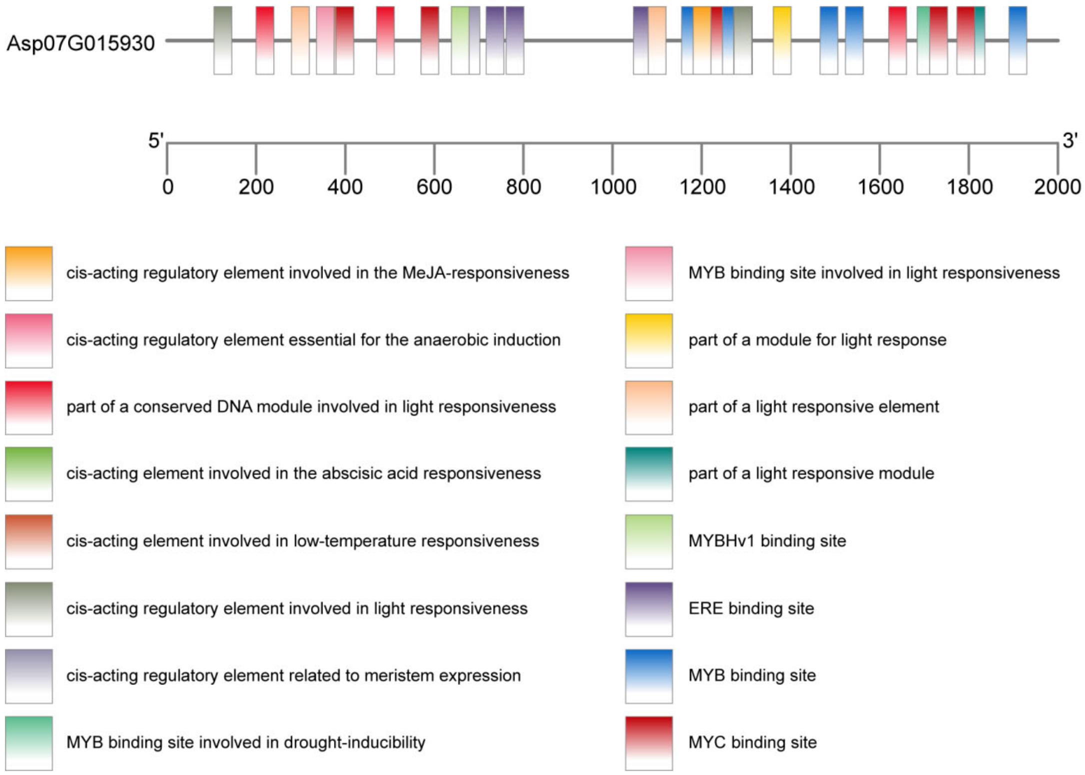

Figure S3. The predicted of cis-acting regulatory elements in the promoter of *A. camelorum* AcABI5.

(a)

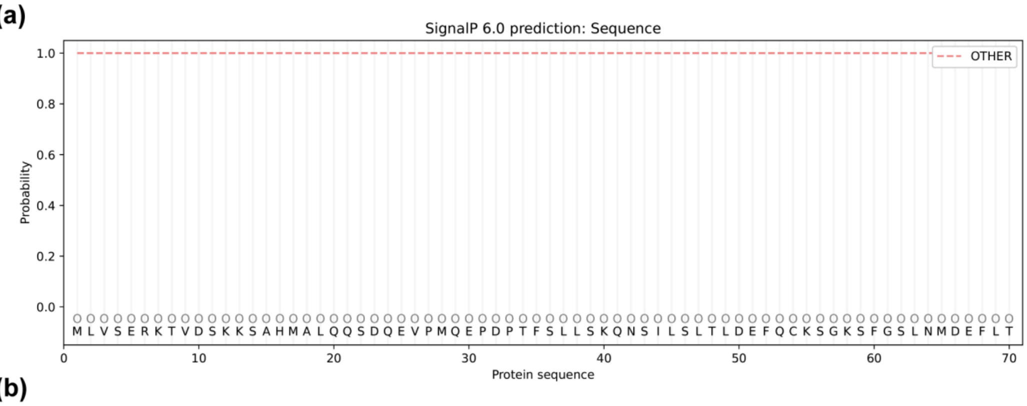

(b)

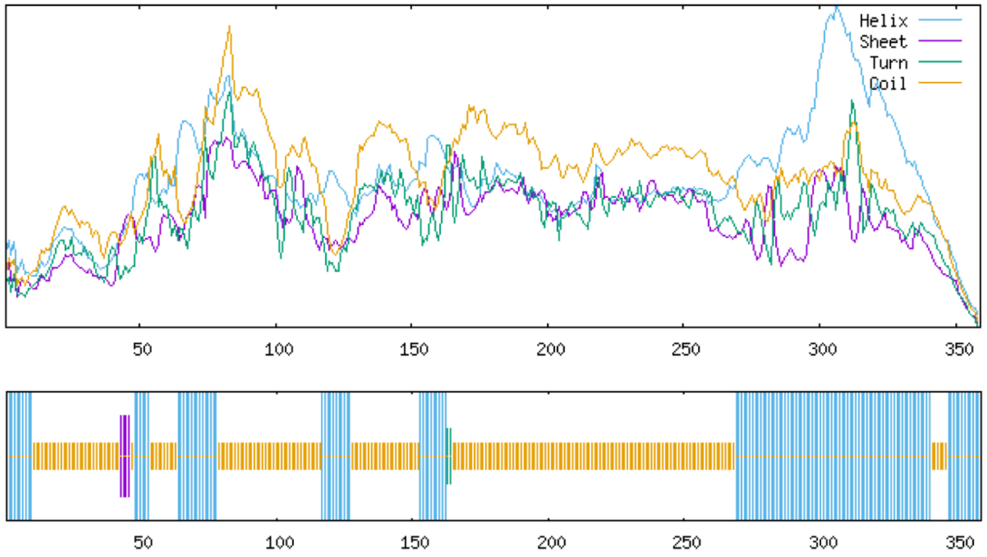

(c)

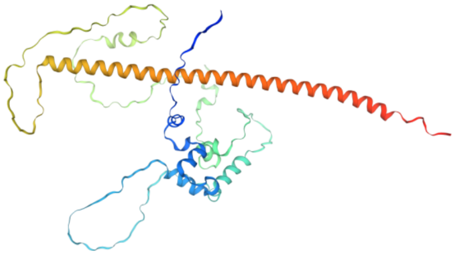

Figure S4. Prediction of (a) signal peptide, (b) secondary structure and (c) tertiary structur of the AcABI5 protein.

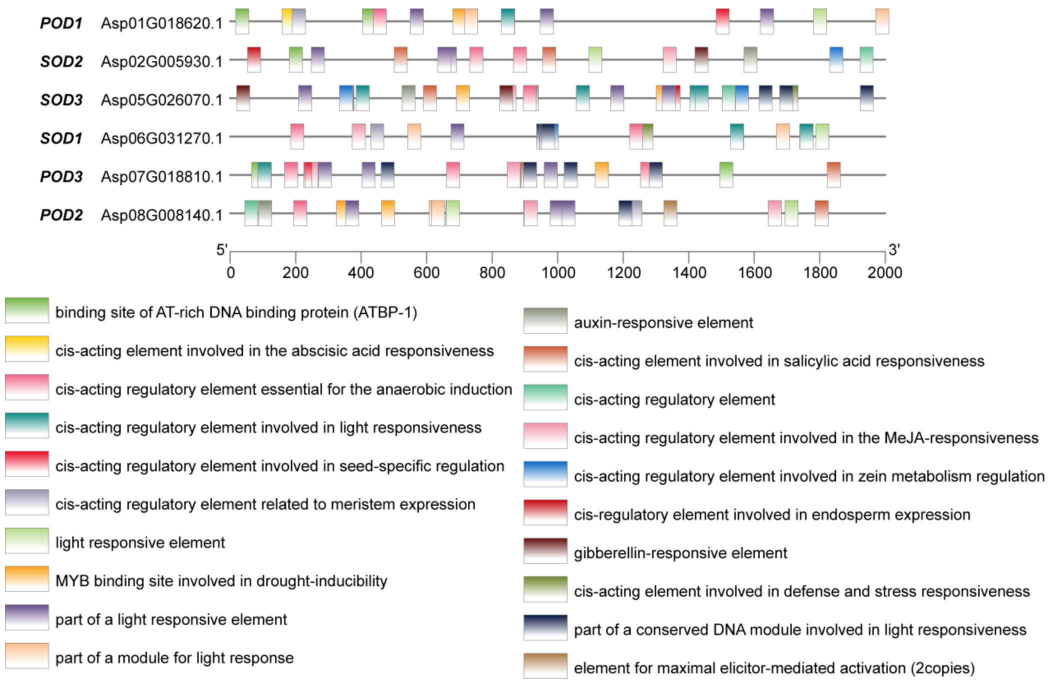

Figure S5. The predicted cis-acting regulatory elements in the promoter sequences of POD and SOD genes from *A. camelorum*.
