# Supplementary material for: De Novo Functional Characterization of AcABI5 Transcription Factor and Its Role in Physiological Responses to Salt Stress in Alhagi camelorum Callus
Source: Int J Mol Sci. 2026 Apr 24;27(9):3812. doi: 10.3390/ijms27093812 (PMC13163759; doi:10.3390/ijms27093812)
Supplement: Supplementary file 1 [file ijms-27-03812-s001.zip › Supplementary Information 2.pdf]

Supplementary Information

Table S1 Effects of different hormone concentrations on callus induction

	Plant growth hormones (mg/l)		Callus induction rate (%)	Callus status	Relative growth rate (%)
	2,4-D	6-BA			
1	0	0	0^{e}	−	$0.95±0.99^{def}$
2		0.2	0^{e}	−	$4.71±0.55^{de}$
3		0.5	0^{e}	D−	$10.02±0.60^{cd}$
4		1	0^{e}	DSG−	$24.81±2.45^{bc}$
5		1.5	0^{e}	DSG−	$37.28±3.60^{b}$
6		2	0^{e}	DSG−	$58.95±9.29^{a}$
7	0.2	0	26^{d}	SB+	$5.11±0.96^{c}$
8		0.2	89^{b}	MB+	$9.53±0.76^{c}$
9		0.5	85^{b}	HG++	$27.72±2.59^{b}$
10		1	100^{a}	HG++	$35.47±3.60^{b}$
11		1.5	0^{e}	DMG+	$54.97±11.98^{a}$
12		2	0^{e}	DMG−	$52.99±13.95^{a}$
13	0.5	0	100^{a}	HG++	$15.02±0.60^{bc}$
14		0.2	100^{a}	HGW++	$28.84±2.37^{b}$
15		0.5	100^{a}	HG++	$42.63±4.07^{a}$
16		1	100^{a}	HG++	$48.83±5.48^{a}$
17		1.5	100^{a}	SGY+	$47.03±4.00^{a}$
18		2	48^{c}	SGY+	$53.56±1.38^{a}$
19	1	0	100^{a}	HG+	$20.21±1.18^{c}$
20		0.2	100^{a}	MG++	$78.52±4.69^{a}$
21		0.5	100^{a}	MGW+++	$92.98±8.63^{a}$
22		1	100^{a}	MGW+++	$60.24±6.46^{b}$
23		1.5	100^{a}	MYW++	$56.49±7.75^{b}$
24		2	100^{a}	MYW++	$35.61±4.35^{c}$
25	1.5	0	100^{a}	HYW+	$21.82±1.89^{d}$
26		0.2	100^{a}	MGY++	$71.05±9.51^{b}$
27		0.5	100^{a}	MGW++++	$110.93±12.52^{a}$
28		1	100^{a}	MG+++	$70.11±5.99^{b}$
29		1.5	100^{a}	MGY++	$53.86±7.74^{c}$
30		2	100^{a}	MG++	$41.76±4.22^{c}$
31	2	0	100^{a}	HBY+	$25.23±2.32^{d}$
32		0.2	100^{a}	HG+	$50.93±5.05^{b}$

Table S1 Effects of different hormone concentrations on callus induction (continued)

	Plant growth hormones (mg/l)		Callus induction rate (%)	Callus status	Relative growth rate (%)
	2,4-D	6-BA			
33		0.5	100[a]	HGW++	83.91±4.20[a]
34		1	100[a]	HGW++	83.36±18.27[a]
35		1.5	100[a]	HG+	64.21±7.45[b]
36		2	100[a]	HG+	46.92±4.69[bc]

[1] Callus status: − indicates no callus growth, + indicates general, ++ indicates good, +++ indicates very good, ++++ indicates excellent. D indicates callus differentiation, S indicates loose texture, M indicates moderate hardness, H indicates compact texture, G indicates green color, W indicates milky white color, Y indicates yellow color, B indicates brown color. Different lowercase letters indicate significant differences by Tukey's test ($P < 0.05$). Data are presented as the mean ± SD ($n = 3$ independent biological replicates).

Table S2 Design of primer sequence for qRT-PCR

Primer Name	Forward Primer (5'–3')	Reverse Primer (5'–3')
ACT1	CTGATAGAATGAGCAAGGAA	CTAAGATAGAGCCACCAATC
EF-1α	TTGACCAAGATTGACAGACGAT	ACGACCAAGAGGAGGATACT
PRP4	CCAGTGTTGAGCAAGAGGCATT	TGGGATAGGCTTTATGACGGGAAT
MS	TATACGGCACCTTGGAAG	GTGTGGAACGATGTTATACC
CLS	GAGCAAGGTTAGCATTCAG	GATGAGAAGAGTAGTCCACTT
PLSH	GCCGCTTACGAAGAGAAC	TAGCAGTGGGAGCAGTTAT
FL4R	AAGATGACTGGCTGGATGTATT	GTGGCATTGTTGGCATAAGAA
ERABI4	GAATCAGCAGCAGAATCAGA	GGTAGTCTTCTTCACTTGTCAA
ABAR	CATAGACGCACCACTACC	CTAACCTCTCCACACTAACC
AP2	GAGGAGGAGGAGGACAAC	CGTCATCATTCCAGTCATCA
ABI5	GCTTACCAGAGTCATCAAGTG	CTACCGTATATGCCTGTCTTCTA
drEBF2	GCTGCTGAAGACAATAACC	CGAAGGAGTAGGAGACATC
AcABI5	CAGATCAAGAGGTGCCAATGC	TGCTGGTTAGGAACTCATCCAT
TRV1-RepL	TGCCATTGGAGGAATCACGCTTA	GGACTCAGATGCCGAATACAGACT
POD1	GGCTCCAACAACAACATTCCTG	GAAGTCTGCTTTGCCATTTCCA
POD2	TGCTTCGCTGCTGAGACTACA	TTGGTCCACCCGTAAGAACAGT
POD3	GGCCGAAGAGATGCAAGAACAG	TGGCAGCGAACATTGAAGTGAG
SOD1	GTCGGTGATGATGGAACTGTGA	AGGATCAGCATGGACAACAACA
SOD2	GGCTCTTCTTCTGCTACCTTCA	AGTGGATATGGTGGTGGCTTCA
SOD3	TCCACCACCAGAAGCATCATCA	TGACCTCCGCCATTGAACTTGA
NbEF1α-1	AGCTTTACCTCCCAAGTCATC	AGAACGCCTGTCAATCTTGG
NbEF1α-2	ATGAACCATCCAGGACAGATTG	CCATACCAGCATCACCATTCTT
NbEF1α-3	AGAGGCCCTCAGACAAAC	TAGGTCCAAAGGTCACAA

.

Table S3 The primer sequence of PCR

Primer name	Primer sequence(5'–3')
pK7WGF2 vector construction	
attB-ABI5-F	AAGCAGGCTGCATGTTAGTTTCTGAGAGA
attB-ABI5-R	GAAAGCTGGGTCCCAGGATGCGCTTATAGG
attB-F	GGGGACAAGTTTGTACAAAAAAGCAGGCTGC
attB-R	GGGGACCACTTTGTACAAGAAAGCTGGGTC
pBI121 vector construction	
pBI121-ABI5-F	ACACGGGGGACTCTAGAGGATCCATGTTAGTTTCTGAGAGAAAAACTGTTG
pBI121-ABI5-R	GCCTATAAGCGCATCCTGGGGTACCATGGTGAGCAAGGG
pTRV2 vector construction	
ABI5-F-EcoRI	GAGTAAGGTTACCGAATTCAATGGACTTGCAAATGC
ABI5-R-BamHI	GTGAGCTCGGTACCGGATCCTTCAGCCAGAATTTGTTTTAGC
pB42AD vector construction	
pB42AD-ABI5-F	AGATTATGCCTCTCCCGAATTCATGTTAGTTTCTGAGAGAAAAACTG
pB42AD-ABI5-R	AAGAAGTCCAAAGCTTCTCGAGTCACCAGGATGCGCTTATAG
pGreenII 62-SK vector construction	
62-SK-ABI5-F	TGGCCATGGAAGGCCGAATTCATGTTAGTTTCTGAGAGAAAAAC
62-SK-ABI5-R	ATGCGGCCGCTGCAGGTCGACCTATAAGCGCATCCTGGTGA
pGBKT7 vector construction	
ABI5-BD-F	gacctgCATATGATGTTAGTTTCTGAGAGAAAAACTG
ABI5-BD-R	gtcgacGGATCCcTCACCAGGATGCGCTTATAGGC
ABI5-BD1-F	gacctgCATATGATGTTAGTTTCTGAGAGAAAAACTG
ABI5-BD1-R	gtcgacGGATCCcTCAGCTCTTCCCACTCTTGC
ABI5-BD2-F	gacctgCATATGTTTGGTTCCCTAAACATGGATG
ABI5-BD2-R	gtcgacGGATCCcTCATCCAAGTGTTTGTTGCT
ABI5-BD3-F	gacctgCATATGGAAATGACTTTGGAGGATTTCT
ABI5-BD3-R	gtcgacGGATCCcTCAGGTAGAAAATCCTATCCCCATTGC
ABI5-BD4-F	gacctgCATATGCAATGTGTGTCTAGCAATGGACTTGC
ABI5-BD4-R	gtcgacGGATCCcTCACCAGGATGCGCTTATAGGC
ABI5-BD5-F	gacctgCATATGATGTTAGTTTCTGAGAGAAAAACTG
ABI5-BD5-R	gtcgacGGATCCcTCAGGTAGAAAATCCTATCCCCATTGC
ABI5-BD6-F	gacctgCATATGCGTAGGATGTTGAAAAATCGAG
ABI5-BD6-R	gtcgacGGATCCcTCACCAGGATGCGCTTATAGGC
ABI5-BD7-F	gacctgCATATGATGTTAGTTTCTGAGAGAAAAACTG
ABI5-BD7-R	gtcgacGGATCCcTCATTGCCTTCGCTCCACTACCAC

Table S3 The primer sequence of PCR (continued)

Primer name	Primer sequence(5'–3')
pLacZi vector construction	
ABRE-F	AATTCACGTGGCACGTGGCACGTGGCC
ABRE-R	TCGAGGCCACGTGCCACGTGCCACGTG
CE3-F	AATTCACGCGTGACGCGTGACGCGTGC
CE3-R	TCGAGCACGCGTCACGCGTCACGCGTG
DPBF-F	AATTCACACGAGACACGAGACACGAGC
DPBF-R	TCGAGCTCGTGTCTCGTGTCTCGTGTG
G-box-F	AATTCCACGTGCACGTGCACGTGC
G-box-R	TCGAGCACGTGCACGTGCACGTGG
sABRE-F	AATTCACGTTTCACGTTTCACGTTTCC
sABRE-R	TCGAGGAAACGTGAAACGTGAAACGTG
Identification of Recombinant Vector	
pK7WGF2-EGFP-F	ACAACCACTACCTGAGCA
pK7WGF2-P17-R	GCCTGCAGGTCACTGGATTT
pK7WGF2-t35S-R	TGCGGACTCTAGCATGGC
pDONR-M13-F	GTAAAACGACGGCCAGTCTT
Egfp-F	GTAAACGGCCACAAGTTCA
Egfp-R	TGTCGGCCATGATATAGACG
M13-F	GTTGTAAAACGACGGCCAG
M13-R	CAGGAAACAGCTATGAC
pTRV2-F	CAGTGCTCTTGGTGTGATTCA
pTRV2-R	AGTGTTCGCCTTGGTAGTAGT
pGBKT7-T7-R	TAGAGGCCCCAAGGGGTTATGC
pGBKT7-F	TCATCGGAAGAGAGTAGT
pB42AD-F	CCAGCCTCTTGCTGAGTGGAGATG
pB42AD-HA-R	AAGCCGACAACCTTGATTGGAG
pLacZi-R	GGACCTAATGTATAAGGAAAG
pLacZi-F	GAATTCGAGCTCGGTACCC
pLacZi-LacZ-R	GCTGGCGTAATAGCGAAG
62-SK-F	GTTGACTGTATCGCCGGAAT
62-SK-R	TCCCTTATCGGGAAACTACTC

Table S4 Analysis of physicochemical properties of AcABI5 protien

Sequence ID	Number of Amino Acid	Molecular Weight	Theoretical pI	Instability Index	Aliphatic Index	Grand Average of Hydropathicity
Asp07G015930	359	40213.27	9.51	56.46	66.57	-0.845

[1] Asp07G015930, i.e., AcABI5.
